# Design and Evaluation of Ergonomic Interventions for the Prevention of Musculoskeletal Disorders in India

**DOI:** 10.1186/2052-4374-26-18

**Published:** 2014-07-01

**Authors:** Somnath Gangopadhyay, Samrat Dev

**Affiliations:** 1 Occupational Ergonomics Laboratory, Department of Physiology, University College of Science & Technology, University of Calcutta, 92 APC Road, Calcutta 700009, India

**Keywords:** Informal sector, Musculoskeletal disorders, Ergonomic interventions

## Abstract

**Background:**

Improper workstation, work procedures and tools are found to be the risk factors for the development of musculoskeletal disorders among the informal sector workers of the developing countries. Low cost ergonomic interventions can effectively improve such adverse conditions.

**Case presentation:**

In the present article some studies related to design interventions in different informal and agricultural sectors were discussed and their efficacies were analyzed. It was observed that with the help of appropriate interventions musculoskeletal disorders were reduced, adverse physiological conditions were improved when awkward postures were corrected and ultimately the organisational productivity was increased.

**Conclusion:**

Proper implementation of ergonomic interventions can ultimately improve the economy of the nation.

## Background

A large number of people are associated with different types of jobs in the informal or unorganized sectors in developing countries. The work units under informal sector share the common characteristics, viz., intense human labor, prolonged work hours, no work-no pay system, irregular work schedule and lack of additional allowances. Consequently they suffer from severe musculoskeletal disorders (MSD), which not only hinder them physically but also enhance their mental agony. Therefore it is imperative to mitigate the onset of musculoskeletal disorders by implementing interventions conducive for specific work activity.

Herberta et al. [1] evaluated the effect of an ergonomic intervention program on the reduction of prevalence and intensity of symptoms of upper extremity work-related musculoskeletal disorders among 36 garment workers performing an operation called spooling. Their results highlighted that 89 percent of the cohort reported pain in either the neck or at least one upper extremity anatomic site prior to the application of intervention. Among subjects reporting pain at baseline, there were significantly decreased pain levels in 10 of 11 anatomic sites after the intervention. This study suggested that introduction of an intervention may diminish musculoskeletal symptoms.

Van der Molen et al. [2] tried to determine the differences in the number of paving stones laid (productivity), task demands, energetic workload, body region discomfort and preference when laying paving stones with or without use of a paver’s trolley. It was observed that the use of a paver’s trolley had no effect on productivity, body region discomfort compared to working without a paver’s trolley. However, six of the eight pavers indicated that, given suitable circumstances, they wanted to use the paver’s trolley.

In another study by Das et al. [3] a typical or conventional workstation for a repetitive drill press operation was evaluated and subsequently redesigned by incorporating the concepts and principles of work design and ergonomics. A methods–time measurement (MTM) analysis was conducted to eliminate unnecessary motions and improve the necessary (work) motions involved in task performance. An experimental investigation was conducted to evaluate and test the redesigned workstation in terms of operator productivity. The increases in quantity and quality output were 22 and 50%, respectively, for the redesigned workstation compared to the typical or conventional workstation. This investigation has demonstrated the beneficial effect of a combined work design and ergonomics approach, especially for the redesign of a workstation.

## Case presentation

### Work related musculo skeletal disorders (WMSDs)

In developing countries nearly 60% of the total working populations are directly involved with different types of jobs in the informal or unorganized sectors. The main reason for this is constant changing economic conditions and easy availability of work forces.

Work related Musculo Skeletal Disorders (WMSDs) are the most prevalent illness among informal sector workers in India. They are the manifestations of the ergonomic hazards and are the leading causes of disability of people during the working years. A majority of these disorders are the results of repeated stress. This may be either due to incorrect design of the equipments used or due to improper design of the workstations. Whatever may be the reason, the net result is the onset of WMSDs.

### Ergonomic interventions

Ergonomic interventions are commonly classified as engineering, administrative or behavioural (or personal). Engineering interventions are engineered or physical manipulations of hazards or routes of exposure to physical hazards. Administrative interventions concentrate on changing the duties or the design of the job such as the introduction of job rotation, enlargement, work cells, or policies. Behavioral interventions focus on the individual worker’s behaviors or capacity. A behavioural (or personal) intervention may focus on increasing fitness or strength, on stress reduction workshops, on improving work methods. Requiring the use of personal protective equipment is a further option and is commonly used in safety and industrial hygiene.

The work-related portion of the injuries and resulting disability is potentially preventable and it is important to identify interventions for reducing work-related musculoskeletal disorders (WMSD).

### Efficacy, effectiveness and cost-effectiveness of implemented interventions

There are many possible outcomes by which ergonomic interventions may be evaluated. Classically these range from efficacy determined under ideal conditions on selected groups in a laboratory or field setting, through effectiveness measured under field conditions on larger groups, to cost-effectiveness or cost-benefit studies.

In the present article, six different sectors were identified. Published and unpublished works of one of two present authors and published works of other Indian researchers on these six different sectors were presented and the efficacy of interventions were analyzed mainly on the basis of productivity and wellness of the physiological conditions of workers (Table 1). On the basis of the response, a strategic plan was drawn on the implementation of interventions for the prevention of WMSDs among these groups of workers. A post intervention study was formulated to find out the effect of the implemented interventions.

**Table 1:** Summary of six studies done in five different sectors

**Authors and sectors**	**Before intervention**								**After intervention**							
	**MSD**	**Physiological parameters**						**Implication of intervention**	**MSD**	**Physiological parameters**						**Evaluation of intervention**
		**HR**	**PHR**	**EE**	**TCCW**	**PCW**	**REP**			**HR**	**PHR**	**EE**	**TCCW**	**PCW**	**REP**	
Gangopadhyay *et.al*[4] Sand Core Making	✓							✓	✓							✓ with increase in productivity
Ghosh and Gangopadhyay [5] Jewellary	✓	✓						✓	✓	✓						✓
Gangopadhyay and Chaudhury. Ergonomic modification of handle of hand saw used by the Indian carpenters (unpublished) Furniture Manufacturing	✓							✓	✓							✓ with increase in productivity
Bhattacharyya and Chakrabarti, [6] Agriculture	✓	✓	✓	✓	✓	✓	✓	✓	✓	✓	✓	✓	✓	✓	✓	✓
Kishtwaria and Rana, [7] Agriculture	✓	✓	✓	✓	✓	✓	✓	✓	✓	✓	✓	✓	✓	✓	✓	✓
Chaturvedi *et.al*[8] Agriculture	✓							✓	✓							✓

HR: Heart Rate; PHR: Peak Heart Rate; EE: Energy Expenditure; TCCW: Total Cost of Cardiac Work; PCW: Physiological Cost of Work; REP: Rating scale of Perceived Exertion.

### Case study I

#### *Implementation of the ergonomic interventions at sand core sector*

In a developing country the improvement of work procedure in any unit can be effectively carried out by means of low cost modifications of existing work process and work station design. This has been successfully performed by Gangopadhyay et al. [4] among the sand core workers.

The sand core making process is a manual job where the workers most often work in awkward postures and suffer from various musculoskeletal disorders primarily affecting the low back region. The existing processes of core making involved some unnecessary steps, which reduces the work rate and increase ineffective time. The modified process involved elimination of these steps and thus reorganize work and enhance productivity.

**Figure 1:**
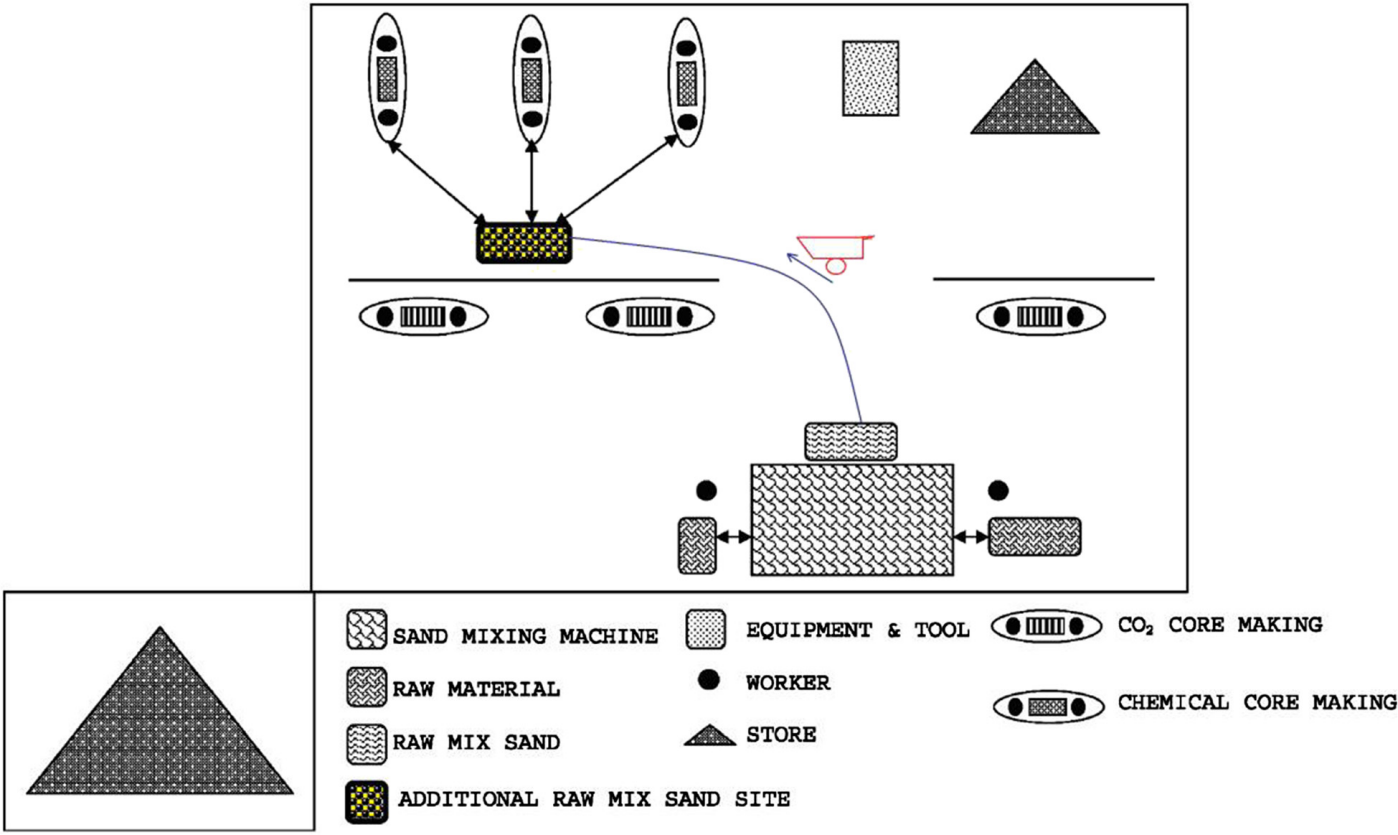
Modified workstation of a sand core making unit.

In the sand core making process, workers had to bring the mixed sand from a long distance repeatedly.A new workstation was designed and implemented where an additional storage site for storing mixed sand was selected near the chemical core making area (Figure 1).

From the modified method study and by designing a new workstation, the total time spent for one core making reduced by 56 seconds from 237 seconds to 181 seconds. As the workers made 50 cores/day, the total time saved was 2800 seconds. Since each core making process took 181 seconds, additional 15 cores could be prepared in a day, with an overall increase in productivity of 30%.

### Case study II

#### *Implementation of the ergonomic interventions at gold smith sector*

**Figure 2:**
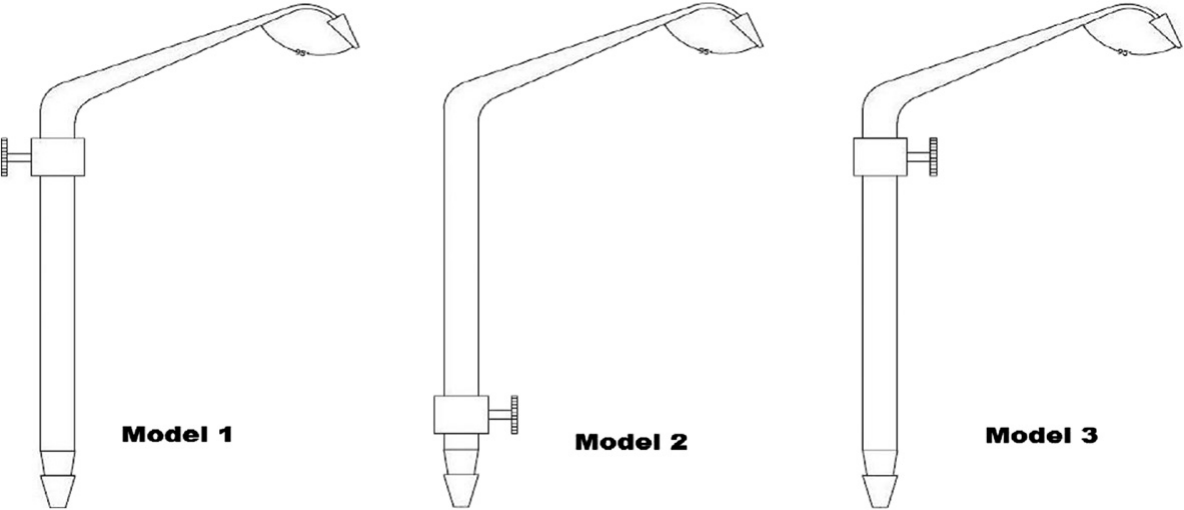
Designed Hand pipes for Gold Smiths (Proposed three types).

Another study involved works on goldsmiths where muscle fatigue and respiratory stress were assessed [5]. A large number of goldsmiths complained of respiratory symptoms in this industry. One of the main activities of the goldsmiths is blowing pipe to heat the gold beads. This continuous blowing out of air from lung produces some pulmonary discomfort. This work habit also increases the fatigue of facial muscles (buccinator, orbicularis oris) at the end of the day.Three different types of hand air pipes were designed (Figure 2).

Finally one type of hand air pipes was selected on the basis of subjects’ preferences. It was observed that the use of mechanical hand air pipe can reduce the physical stress on goldsmiths. After work with mechanical hand pipe, physical stress as heart beats of the goldsmiths were significantly reduced (77.13 beats/min) than after work heart beats (82.04 beats/min) with blow pipe.

### Case study III

#### *Implementation of the ergonomic interventions at carpentry sector*

**Figure 3:**
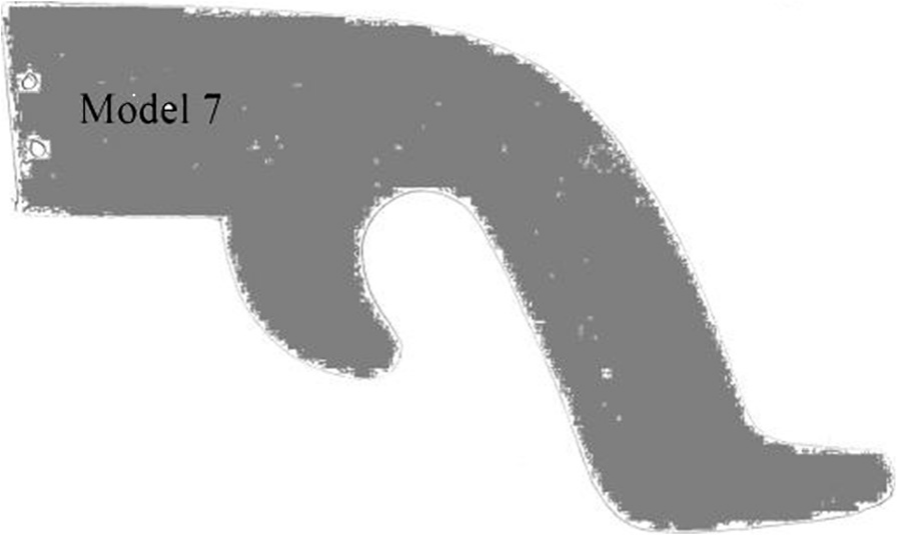
Selected Handsaw handle (M7).

Hand tools should be designed according to the preference on the basis of comforts of the users. Tools should be shaped to avoid wrist deviations, allowing the hand and fore arm to remain in linear condition during forceful grip. On this concept nine different handles of handsaws were designed.Among these saws, handle of M7 was pistol handle shaped which helps in avoiding wrist deviations and can reduce the fatigue of hand muscles (Figure 3). This handle also has a guard to protect fingers to come in contact with the saw blade.

M7 could improve the carpenter’s health and reduce injury during work, which could be treated as the increment of productivity, safety and health of carpenters.

### Case study IV

#### *Implementation of the ergonomic interventions at agriculture (Tea planting) sector*

The Tea Industry is one of the largest employers of women nearly 51% of the total workforce in Assam. Many of the activities, especially the plucking activity performed by the workers demand a high level of physical effort because of repetitiveness and static awkward posture, leading to early fatigue and work related musculoskeletal problems.

To improve the situation and to reduce the work related discomfort an ergonomically light weighted and high capacity basket was introduced [6], which was based on the relevant anthropometric measurements and the basket as such fits the back of the workers so that it does not shift its position.

By using existing basket workers reported musculoskeletal problems at different parts of the body. But after the use of modified basket a significant reduction in discomfort at different body parts was observed.

Average working and peak heart rate of workers was found to be 96.26 beats/min and 100.6 beats/min respectively while plucking with improved cane basket, while both were found to be increased to 99.37 beats/min and 106.9 beats/min while using the existing bamboo basket. In case of average and peak energy expenditure with improved basket was 7.07 kJ/min and 6.57 kJ/min, both were found to be increased to 8.27 kJ/min and 7.27 kJ/min while using existing bamboo basket. Similarly physiological cost of work (PCW) was also increased to 22.58-beats/min with existing basket in comparison to improved cane basket, which resulted to 21.74 beats/min.

### Case study V

#### *Implementation of the ergonomic interventions at agricultural sector during weeding activity*

This study was carried out on hill farm, where women are mainly associated with weeding activities with the help of traditional tools. The study was undertaken to ascertain the health status and ergonomic cost of weeding with existing and improved technology. The existing tools were improved as per anthropometric data and physical fitness levels of hill women. Three weeding tools were improved and introduced after modification viz. weeders, kutla and small hoe. These tools were made light in weight to ease the operation with suitable length, sharp inner edges and convenient handles [7].

The comparative assessment between uses of traditional vs. improved tools in both the states showed better results in terms of heart rate values. The findings of ergonomic assessment for the traditional and improved tools under the study showed that heart rate values (93 beats/min and 103 beats/min) were more than acceptable limits while they were weeding with the traditional tools.

### Case study VI

#### *Implementation of the ergonomic interventions at agricultural sector during activities with power tiller*

This agriculture study highlighted the problems of workers who used the power tiller for different activities in the farm field [8]. The high level of vibration from the power tiller was transmitted from the handle to hands, arms and shoulders caused early fatigue to the operator. Three materials were used for intervention development to reduce vibration magnitude. They were polyurethane, rubber and combination of polyurethane and rubber. It was found that the maximum vibration reductions were achieved with the rubber in all three operational conditions.

It was observed that the working heart rate was higher for rota-tilling than the tilling with cultivator and transportation. The maximum value of working heart rate for rota-tilling was 141.15 beats/min without interventions. With the help of interventions the working heart rate reduced to 131.56 beats/min, which is a significant reduction. With the application of interventions the Body Parts Discomfort Scale (BPDS) also reduced for each operating condition mainly observed in transportation activity by reduction of BPDS from 36.06 to 22.27 with the application of interventions, indicating that vibrations are major contributing factor for the discomfort score.

## Conclusion

It is concluded from the evaluations of results of six different studies that workers in the unorganized and agricultural sectors are compelled to work with maximum amount of physical effort and minimum amount of safety. Consequently these people perform strenuous manual tasks for prolonged periods and suffer from musculoskeletal disorders afflicting different body parts. For them ergonomic-interventions are the best solutions for the prevention of Work related Musculoskeletal Disorders. However the underneath reasons for concern is that most of the interventions are improperly designed and the most significant aspect that deserves special mentioning is lack of maintenance. Despite the constraints, the workers of the unorganized or informal sectors of India mainly sand core making workers, gold smiths carpenters are highly benefited by ergonomic interventions as modified workstations and newly designed tools.

## Consents

Written informed consents were taken from the subjects before experiments and for publication of this report.

## Competing interests

The authors report no conflict of interests.

## Authors’ contributions

SG contributes towards the initiation of the concept of the study and critical revision and evaluation of the manuscript. SD renders the technical support and, drafting of the manuscript. Both authors read and approved the final manuscript.
